# Protocol for the Development of a Behavioral Family Lifestyle Intervention Supported by Mobile Health to Improve Weight Self-Management in Children With Asthma and Obesity

**DOI:** 10.2196/13549

**Published:** 2019-06-24

**Authors:** David Fedele, Robert Lucero, David Janicke, Mutasim Abu-Hasan, Elizabeth McQuaid, Jon Moon, Andrea Fidler, Tanya Wallace-Farquharson, David Lindberg

**Affiliations:** 1 Department of Clinical & Health Psychology University of Florida Gainesville, FL United States; 2 Department of Family, Community, and Health System Science University of Florida Gainesville, FL United States; 3 Center for Latin American Studies University of Florida Gainesville, FL United States; 4 Department of Pediatrics University of Florida Gainesville, FL United States; 5 Departments of Psychiatry and Human Behavior and Pediatrics Brown University Providence, RI United States; 6 MEI Research, Ltd Edina, MN United States; 7 Department of Biobehavioral Nursing Science University of Florida Gainesville, FL United States; 8 Department of Statistics University of Florida Gainesville, FL United States

**Keywords:** asthma, obesity, child, family, program development

## Abstract

**Background:**

Asthma is the most common chronic childhood illness and is a leading cause of emergency department visits in the United States. Obesity increases the risk of poor health outcomes, reduced quality of life, and increased health care expenditures among youth with asthma. Weight loss is crucial for improving asthma outcomes in children with obesity. Our study team developed the Childhood Health and Asthma Management Program (CHAMP), a 16-session behavioral family lifestyle intervention (BFI) for school-age children with asthma and obesity and evaluated CHAMP in a randomized controlled trial compared with attention control. There were medium effect sizes favoring CHAMP for changes in body mass index z-scores, asthma control, and lung function among completers (ie, those who attended ≥9 of 16 sessions). Despite high rates of satisfaction reported by families, attendance and trial attrition were suboptimal, which raised concerns regarding the feasibility of CHAMP. Qualitative feedback from participants indicated 3 areas for refinement: (1) a less burdensome intervention modality, (2) a more individually tailored intervention experience, and (3) that interventionists can better answer health-related questions.

**Objective:**

We propose to improve upon our pilot intervention by developing the Mobile Childhood Health and Asthma Management Program (mCHAMP), a nurse-delivered BFI, delivered to individual families, and supported by a mobile health (mHealth) app. This study aims to (1) identify structural components of mCHAMP and (2) develop and test the usability of our mCHAMP app.

**Methods:**

Participants will be recruited from an outpatient pediatric pulmonary clinic. We will identify the structural components of mCHAMP by conducting a needs assessment with parents of children with asthma and obesity. Subsequently, we will develop and test our mCHAMP app using an iterative process that includes usability testing with target users and pediatric nurses.

**Results:**

This study was funded in 2018; 13 parents of children with asthma and obesity participated in the needs assessment. Preliminary themes from focus groups and individual meetings included barriers to engaging in health-promoting behaviors, perceived relationships between asthma and obesity, facilitators to behavior change, and intervention preferences. Participatory design sessions and usability testing are expected to conclude in late 2019.

**Conclusions:**

Outcomes from this study are expected to include an mHealth app designed with direct participation from the target audience and usability data from stakeholders as well as potential end users.

**International Registered Report Identifier (IRRID):**

DERR1-10.2196/13549

## Introduction

### Background

Asthma affects more than 8% of youth and is a leading cause of emergency department visits in the United States [1-3]. Recent evidence indicates that pediatric asthma and obesity are interrelated, with heightened weight status modifying asthma course [4]. Indeed, obesity places children with asthma at an increased risk for poor health outcomes, reduced quality of life, and increased health care expenditures [5-11]. Weight loss is critical for improving asthma outcomes in individuals with obesity [12]. Behavioral family lifestyle interventions (BFIs) are the most effective weight self-management interventions for children with obesity [13,14]. BFIs, grounded in social cognitive theory [15-17], help families acquire self-management skills to better regulate their dietary intake and physical activity behaviors, ultimately leading to reduced child adiposity [18,19].

Our study team recently developed the Childhood Health and Asthma Management Program (CHAMP), a tailored BFI for children with asthma and obesity [20]. Over a 4-month period, parent and child dyads were asked to attend 12 group-based sessions and 4 individual family sessions. Parent and child sessions occurred on weekday evenings, ran concurrently, and were designed to last approximately 90 min. Each session adhered to the following general structure: (1) a review of parent and child progress, (2) skills training and implementation, and (3) goal setting and encouragement. Most families who participated in the trial identified as African American (67%, 16/24) and reported an annual family income of less than US $35,000. Preliminary pilot testing comparing CHAMP with a rigorous attention control produced mixed findings. CHAMP earned high participant satisfaction ratings and medium effect sizes favoring CHAMP for changes in body mass index (BMI) z-scores, asthma control, and lung function among completers [20]. However, consistent with other BFIs [21-23], intervention session attendance was challenging as families reported barriers to attending in-person groups, including travel distance, and scheduling conflicts. Furthermore, qualitative feedback indicated 3 clear areas for refinement: (1) a less burdensome intervention modality, (2) a more individually tailored intervention experience, and (3) interventionists that can better answer health-related questions.

Mobile health (mHealth) technologies are ubiquitous across sociodemographic strata [24]. Moreover, mHealth has emerged as an effective platform that can improve diet and physical activity in youth with obesity, facilitate the delivery of personalized intervention content, and can address some of the time- and access-related barriers faced by families of children with asthma and obesity [25]. In addition, nurses have the requisite knowledge and skill set to educate, motivate, and successfully assist families to use behavioral weight self-management skills to facilitate health behavior change [26,27]. Their credibility with families and widespread integration into pediatric health care and community settings across the country increase the promise of downstream scalability. We posit that a nurse-delivered BFI, delivered to individual families and supported by mHealth, is the right approach to overcome attendance challenges associated with family and work schedules [26-29] and will be responsive to participant feedback from our pilot trial.

### Study Aims

We propose to leverage lessons learned from our pilot trial to develop Mobile Childhood Asthma Management Program (mCHAMP), a nurse-delivered BFI supported by mHealth**.** To achieve this purpose, we will do the following:

1. Identify the structural components of mCHAMP using a valid collaborative approach for adapting evidenced-based interventions, known as the Assessment Decision Administration Production Topical Experts Integration Training Testing (ADAPT-ITT) model [30]. The first 2 phases of the ADAPT-ITT model, assessment and decision, will be used to guide the adaptation process. We will conduct a needs assessment with parents (n=20) of children with asthma and obesity to produce the principal components of mCHAMP. The modified intervention protocol will reflect and be responsive to the needs of families of children with asthma and obesity.
2. Develop and test our mCHAMP mobile app to enable a nurse-led BFI for children with asthma and obesity. Using an iterative process, we will develop (n=10) and conduct usability testing (n=10) of the mCHAMP app with parents of children with asthma and obesity [31]. We will then gather provider feedback on clinical implementation from pediatric nurses (n=5).

## Methods

### Study Design

We will use the ADAPT-ITT model to modify our existing CHAMP protocol and create the mCHAMP app. Table 1 (below) presents the timeline for activities to achieve the study aims.

**Table 1:** Twelve-month project timeline for study activities.

Activity	Months											
	1	2	3	4	5	6	7	8	9	10	11	12
Study preparation	✓^a^	—^b^	—	—	—	—	—	—	—	—	—	—
Recruitment/needs assessment	✓	✓	—	—	—	—	—	—	—	—	—	—
Design sessions	—	✓	✓	—	—	—	—	—	—	—	—	—
mCHAMP^c^ tool development	—	—	✓	✓	✓	✓	✓	—	—	—	—	—
Usability testing/provider feedback	—	—	—	—	—	—	—	✓	—	—	—	—
Data analysis	✓	✓	✓	—	—	—	—	✓	✓	—	—	—
mCHAMP final refinement	—	—	—	—	—	—	—	—	—	✓	—	—
Dissemination	—	—	—	—	—	—	—	—	—	—	✓	✓

^a^Activity planned for corresponding month.

^b^Not applicable.

^c^mCHAMP: Mobile Childhood Health and Asthma Management Program.

Institutional review board approval and Information Technology security clearance was sought and initiated during the application review period. Any remaining study preparations will be completed in month 1, including hiring graduate research assistants and setting-up a participant reimbursement account. Participant recruitment and data collection will occur from months 1 to 3. Ongoing qualitative data analysis will occur in months 1 to 3. The research team will collaborate with MEI Research, Ltd during months 3 to 7 to develop the mCHAMP app. mCHAMP usability testing with parents and feedback from registered nurses will occur during month 8. Analysis of usability data and synthesis of registered nurse feedback will be conducted during months 8 and 9. MEI will make final refinements to the mCHAMP app based on usability and feedback results.

### Participants

#### Inclusion and Exclusion Criteria

Caregiver participants must be parents or legal guardians of a child who (1) is between the ages of 6 and 12 years, (2) with a physician-verified current diagnosis of asthma for ≥6 months, (3) with a BMI ≥85th percentile for biological sex and age as published by the Centers for Disease Control and Prevention [32], and (4) lives in the home with the parent. Parents must also speak and read English. There is no BMI requirement for participating parents/legal guardians. Child asthma, height, and weight will be verified by electronic medical record if recruited from the medical system. Parent report of child’s height and weight will be used for individuals recruited outside the medical system. Parents will be excluded if they have a significant cognitive impairment or developmental delay that interferes with study completion. Nurse participants must be registered nurses who (1) provide care for children with asthma and (2) have worked in that capacity for 1 or more years.

#### Recruitment

Parent participants will be recruited from a pediatric pulmonary clinic, as well as local advertisements distributed through community organizations, physician offices, and schools. When feasible, research staff will be available to briefly meet with potential participants during a scheduled clinic visit. In coordination with clinic staff, research staff will meet with interested participants to provide a study overview, complete in-person screening for eligibility, and invite participation. In the event that a family is unable to complete screening during a clinic visit, clinic or research staff will request permission for a member of the study team to contact patients for screening and document that consent in writing. A member of the study staff will then call interested participants to provide a study overview and invite participation. Nurse participants will be recruited from flyers and through professional networks.

#### Enrollment/Informed Consent

Participant enrollment date and the capacity of each study phase will determine which tasks (eg, focus group, design sessions, or usability testing) parents are asked to participate in. During informed consent, the available study tasks will be explicitly stated, and parents will have the option to select which task(s) they want to participate in. Participation in 1 research task does not preclude participation in additional research tasks.

All participants will be provided written informed consent regarding risks, benefits, confidentiality, incentives, and the name and phone numbers, so they may call if they have additional questions. We will receive both verbal and signature consent from participants to participate. Finally, additional informed consents will be obtained to audio-tape meetings for quality control purposes. Participants will be informed that the tapes will be used to transcribe their responses for full review and will not be used for anything other than research purposes.

#### Retention Plan

We did not have a well-defined retention plan for CHAMP. Thus, we developed the following retention strategies given the previously noted attendance difficulties and attrition in CHAMP. At the start of the study, we will aim to engage participants in a discussion surrounding the importance of their contributions to research. The study’s requirements will be clearly and concisely conveyed to the participant. We will also discuss the impact of dropouts and the importance of returning for any additional sessions, consider barriers that may impede participation, and work together to develop strategies to overcome the identified barriers. We will obtain several phone numbers (home, cell, and work) for each family to decrease the likelihood of attrition owing to changing residences, phones, or phone numbers. During this initial visit, we will also record the best time to call families and whether it is acceptable to call them at work. A tracking system will also be implemented to track participant sessions and log all contact attempts.

The day before the session, participants will be contacted via their preferred method of communication to review visit reminders and to confirm the appointment. For participants who do not answer, a voicemail will be left listing the date/time of the scheduled visit as well as instructions to return our call to confirm the appointment. Participants who miss a session will be called 5 min after the anticipated start time of the session. If a participant is still unable to be reached, a voicemail will be left asking the participant to call our study staff. If participants do not return the call within 2 days, the reestablishment of contact procedure detailed in the following paragraph will be implemented.

When a participant cannot be reached, they will be called 5 times to establish contact; calls will be made at varying times of day and days of the week. After 3 contact attempts, a secondary phone number will be called. Study staff will identify themselves, explain the reason for the call, and confirm the participant’s current contact information. If no new phone numbers are obtained, the secondary contact will be asked to pass a message on to the participating family. Participants who cannot be contacted will be sent a letter reminding them of upcoming sessions and asking them to contact study staff.

### Aim 1: Identify the Structural Components of Mobile Childhood Health and Asthma Management Program

In line with the ADAPT-ITT model [30], we will conduct assessment and decision phases using feedback from parents of children with asthma and obesity to design the mCHAMP.

#### Phase 1: Needs Assessment

We will recruit 20 parents of children with asthma and obesity to participate in 4 focus groups (between 4 and 8 parents per focus group). Each parent will participate in only 1 focus group. In the event that scheduling in-person focus groups is nonviable (eg, unable to identify shared availability across participants in the upcoming 3 weeks), we will conduct individual meetings with participants. Audio of all focus groups and individual meetings will be recorded. Focus groups and individual meetings will take place at the University of Florida (UF), a convenient community location, or over a video chat interface that enables compliance with the Health Insurance Portability and Accountability Act of 1996 (Zoom). Focus groups and individual meetings will last approximately 1 hour, and parents will be compensated US $50 for their participation.

The purpose of these groups and individual meetings will be to identify what structure and components of our existing CHAMP protocol are most salient to individual families of children with asthma and obesity. Participants will complete a demographic questionnaire and technology use questionnaire. Then, we will begin by explaining the purpose of the focus group and exploring broad question categories about asthma and weight self-management, which are as follows:

1. What changes, if any, have you or your family made because of your child’s asthma?
2. What is your family’s experience with trying to balance taking care of asthma and making healthy choices?
3. What things get in the way of your family being more active or eating healthier?

Next, we will introduce the intervention in its current form and seek feedback as follows:

1. What type of intervention content (eg, behavior monitoring, skills training, and goal setting) would be most feasible and helpful?
2. How can we be most helpful in teaching and practicing these skills with families?
3. How might a smartphone app be used to help families learn new skills, set goals, keep track of behaviors, and problem-solve things that get in the way?
4. How would you prefer to be in contact with a nurse interventionist (ie, face-to-face, telephone, and video conferencing)?
5. How many contacts do you think would allow you to learn the content?

Finally, we will summarize the main points of the discussion, ask for any comments or corrections, and ask whether we missed anything in our discussion.

Recordings of focus groups and individual meetings will be transcribed verbatim by a professional transcription service. We will enter transcribed files and expanded field notes into NVivo (QSR International). We will code and aggregate interviews using a theoretical thematic analysis approach to developing themes [33-35] and double-check for inconsistencies in the transcripts against the audio recordings. Our theoretical thematic analysis approach will use an a priori theoretical framework guided by social cognitive theory. We will mark comments identified to represent discrete thoughts or themes using a semantic analysis and use an essential realist approach to arrive at themes [33]. These patterns or themes will comprise the initial set of categories. DF and DJ and research staff will then recode the data using these categories. Any disagreements will be resolved based on consensus or a two-thirds agreement. We used empirical guidelines to establish our initial sample size goals [36]. We will recruit additional study participants if saturation is not achieved with the planned participant enrollment. We will organize major themes into summary tables to inform the decision phase.

#### Phase 1: Decision

We will develop an intervention protocol draft for mCHAMP using data from phase 1 and the collective expertise of the research team. Within the framework of BFIs for weight self-management, the study team will address a set of core questions related to key content and activities. When differences of opinion occur, we will apply a two-thirds consensus method to adopt the research team member’s input into the intervention protocol draft.

##### Mobile Childhood Health and Asthma Management Program Content

The core questions related to key content are as follows:

1. What are the core behavioral weight self-management skills that should be provided as part of the intervention?
2. What content from our existing CHAMP protocol should be further adapted to address the needs of these families?
3. What additional content could we add on the basis of the needs of families of children with asthma and obesity?
4. How do we best position pediatric nurses to serve as interventionists?
5. What should be the overall number and type (face-to-face vs phone) of intervention contacts?

##### Mobile Childhood Health and Asthma Management Program Delivery

The core questions related to activities are as follows:

1. What is the best modality to deploy specific intervention content and tasks (eg, in-person, phone, or via the mCHAMP app)?
2. How can we best leverage the mCHAMP app to overcome attendance and participation barriers we encountered in our pilot study?

### Aim 2: Mobile Health Weight Self-Management Tool Development

**Figure 1:**
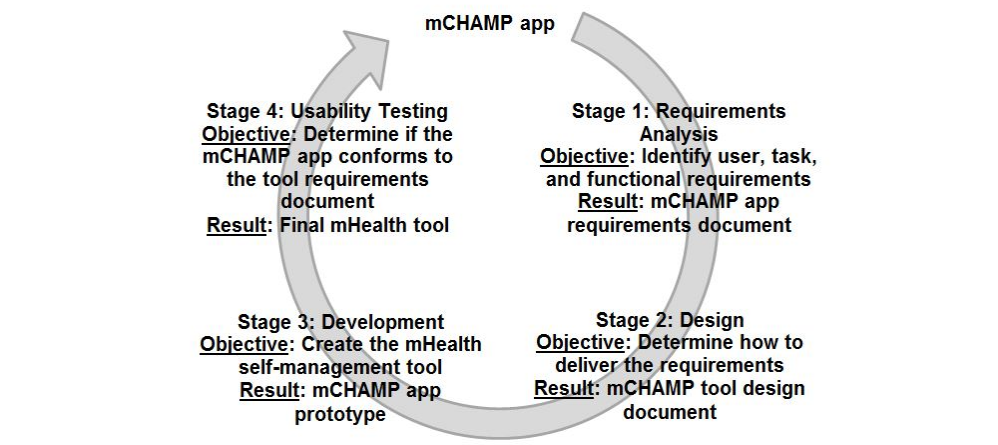
Development process for the Mobile Childhood Health and Asthma Management Program (mCHAMP) app. mHealth: mobile health.

Figure 1 shows the life cycle for developing the mCHAMP app. The result will be an mHealth tool that will be integrated with the revised mCHAMP protocol for families of children with asthma and obesity. One of the lead authors (RL), who is an expert in user-centered design including usability testing, will direct and participate in the implementation of the consumer-centered participatory design (C^2^PD) approach that will be used to develop mCHAMP tool [31]. This approach has been used by Dr Lucero in other consumer health domains such as fall prevention among community-dwelling older adults and family caregiving of persons living with dementia to develop both personal computer- and app-based self-management tools [37-39].

#### Stage 1: Requirements Analysis

The C^2^PD approach will be used to elicit user requirements [31]. Study participants and investigators focus on 4 specific domain requirements: (1) user characteristics and needs, (2) system content and functions, (3) content function and use, and (4) representation of content and function.

For this phase, we will recruit 10 parents of children (aged between 6 and 12 years) with asthma and obesity to participate in a series of 3 participatory design sessions (PDSs). There will be 2 separate design groups (n=5 per group), with each group meeting 3 times. Parents will be compensated US $50 for their participation in each design session (up to US $150 total). Participants will complete a demographic questionnaire and technology use questionnaire. Design sessions will be audio-recorded and will take place at UF, a location in the community, or over Zoom. In the event that scheduling group sessions is not feasible, we will conduct individual meetings with participants. Participant time commitment and compensation will remain consistent regardless of design group method.

After each session, DF, RL, and DJ will debrief to note important system requirements. Examples of design session activities that have been implemented in our previous research include the following: interactive multiple-choice selection using visual cues, low-fidelity prototyping, interactive observation, and participant-designed 2-dimensional interfaces, photo elicitation, and constructing collages [37]. *PDS 1, user assessment,* is the process of identifying user characteristics, such as technology expertise and skills (eg, use of smartphone and tablet), comfort in interacting with technology, educational background, and preferences between visual and text to communicate information. *PDS 2, functional assessment*, is the process of identifying system goals of mCHAMP. Parents may find it necessary for the mCHAMP app to contain a function that can be used to facilitate self-monitoring related to caloric intake. *PDS 3, representation and task assessment*, involves identifying preferred task structures and procedures, such as input and output formats of information and communication flow (ie, representation) and ways of searching, entering, accessing, or retrieving information (ie, task) from the app.

Recordings of design sessions will be transcribed verbatim by a professional transcription service. NVivo 11 software (QSR International) will be used to analyze the data. A descriptive content analysis will be conducted for each session to generate a list of user, functional, and representation and task requirements. Two members of the research team (Fedele and Lucero) will read the transcripts to identify recurring or specific content. They will meet to review, clarify, and draw consensus on emerging content with input from the research team. The output of the analysis will be a set of requirements for the design of the mCHAMP app. On the basis of the requirements for the mCHAMP app, the research team will draft a requirements or design document to guide prototype development.

#### Stage 2: Design

We anticipate that the mCHAMP app used to support our BFI could include several components to support our revised intervention protocol (see Table 2).

**Table 2:** Potential mCHAMP intervention components.

Potential app component	Potential Functionality
Weight self-management education	Providing information on American Academy of Pediatrics recommendations for dietary, physical activity, and screen time; discussing how to build a home environment that promotes healthy dietary habits and engagement in physical activity (eg, stimulus control); providing education regarding the importance and safety of exercise for children with asthma and collaboratively brainstorming ways to increase physical activity in their family and community; discussing the importance of regularly self-monitoring dietary habits and physical activities; and providing feedback on behavioral parenting strategies (eg, positive reinforcement, differential attention, and contingency management) to overcome child resistance.
Remote monitoring	Ability for family and nurse to monitor the child’s dietary habits (eg, servings of fruits and vegetables and sugar sweetened beverages), screen time, number of steps, nonstep-based activities (eg, swimming), and the child’s weight.
Goal setting	Information on how to complete structured goal setting and action planning for the week using a SMART^a^ goal framework.
Communication with nurses	Ability to send nurses questions regarding intervention content, review goal setting, and remote monitoring of weight self-management behaviors.

^a^SMART: Specific, Measurable, Attainable, Relevant, Time-Bound.

#### Stage 3: Development

Existing technology provided by MEI Research, the technology partner, will be leveraged to develop mCHAMP through an iterative process [40]. This technology includes PiLR ecological momentary assessment (EMA) that is part of the patented PiLR Health system. PiLR EMA is a smartphone software that can deliver interactive, real-time surveys and intervention content that we are able to configure at the individual or group level. PiLR EMA compiles to native Android and iOS software for mobile devices to ensure that it functions in offline situations to record sensor data and provide assessments and content. PiLR EMA is supported and operated remotely by the PiLR system. PiLR is a cloud-based platform to collect, store, process, and report on objective behavioral data from surveys and multiple sensors. Real-time surveys and intervention content are designed through a Web portal and can be delivered by time, initiated by the participant, or initiated by sensor-based triggers. We will work with MEI Research to customize PiLR EMA to create an app specifically for mCHAMP. We will provide oversight to ensure fidelity of the design approach and adherence to usability principles.

#### Stage 4: Usability Testing

Once the mCHAMP app prototype has been developed, we will conduct usability evaluations with 10 parents of children with asthma and obesity. Usability testing will take place at UF. Usability testing will be conducted individually with each parent and will last approximately 1 hour. Parents will be compensated US $50 for their participation. Participants will complete a demographic questionnaire and technology use questionnaire. We will focus on measures related to the user as well as functions, representation of information, and tasks in the app. The usability guide will consist of a series of scenarios to test simple and complex features of the mHealth tool. Participants will be asked to think aloud as they use the prototype. Their verbalizations and the researcher’s prompts and clarifications will be audio-recorded. We will assess the usability of the app via the following methods:

##### Usefulness

A functional analysis will inform the potential usefulness of the mCHAMP tool. Features wanted by parents will be compared with the following: (1) all of the features and content in the tool, (2) the features and content wanted by parents but not in the tool, and (3) the features and content included in the tool (ie, designer’s model) but not in the user model (ie, parents) *.* These comparisons will facilitate identifying what features and content should be included in the mCHAMP tool based on the needs of parents.

##### Usability

A representation and task analysis will evaluate how usable the mCHAMP tool is for families of children with asthma and obesity. Representation analysis will be conducted to evaluate how usable the tool is for task completion. Specifically, the analysis will evaluate the ability of parents to recognize the built-in cues of the tool to support completing tasks. The more cues are recognized and used, the more usable the tool should be for parents. Proportions of cues will be calculated for each participant to characterize how well the tool can support end users. Task analysis will evaluate how usable the tool is. Two criteria will be used: (1) learnability: the ease of learning and relearning and (2) efficiency: the effort used to accomplish a task. *Learnability* will be characterized based on the count of errors and the count of hints and prompts needed to recover from errors faced by parents. *Efficiency* will be described by reporting the number of steps and the duration of time needed to accomplish each task. If the tool is difficult to learn and**/**or inefficient for any participant, we will identify problem tasks and modify the tool.

##### Satisfaction

The Post-Study e-Health Usability Questionnaire (PSHUQ [41]) will be used to evaluate parent satisfaction with the mCHAMP tool at the end of the usability testing session. The PSHUQ is an 18-item Likert-type scale ranging from 1 (*strongly agree*) to 7 (*strongly disagree*). The PSHUQ is composed of 2 subscales: (1) system usefulness—ease of completing task and (2) system quality—satisfaction with the quality of information and interface. The mean, SD, and variance for each of the individual items from the PSHUQ tool will be computed. An overall satisfaction score will be obtained by calculating the average scores for each parent across both subscales. Boxplots will be constructed for each of the individual items as well as the aggregate satisfaction to detect for outliers. If the aggregate satisfaction rating is greater than 3.5 on a scale of 1 (ie, positive rating) to 7 (ie, negative rating), or if any outliers are detected on any individual scale, we will review individual PSHUQ scores and consider modifications to the tool.

#### Phase 4: Provider Feedback

We will conduct semistructured interviews with pediatric nurses who provide care to 6- to 12-year-old children with asthma (n=5). We will explore what they perceive as potential barriers and facilitators of delivering our proposed mCHAMP intervention for their patient population. Nurses will complete informed consent before participating. Before the interview, nurse participants will have access to a prototype of mCHAMP, and study staff will explain the project and its goals. Specific questions include the following:

1. Does mCHAMP capture intervention content that would be useful for your patients?
2. How could we engage nurses in using the mCHAMP app and delivering the BFI for their patients?
3. How would the mCHAMP app and BFI fit in your typical clinic workflow?
4. What, if any, modifications could we make to mCHAMP to reduce barriers to implementation?

Individual semistructured interviews will be scheduled to last approximately 1 hour. Male and female providers of any race and ethnicity will be invited to participate in the interview. Interviews will be conducted in-person and will be audio-recorded. Participants will also complete a demographic questionnaire and technology use questionnaire. Nurses will be compensated US $100 for their participation. Data generated from these interviews will inform future refinements of mCHAMP in advance of future research directions (eg, pilot efficacy testing).

## Results

This study was funded in 2018, and recruitment started in September 2018. At this time, 13 parents of children with asthma and obesity have been recruited from an outpatient pediatric pulmonary clinic and consented for participation in the needs assessment; 7 parents participated in focus groups, each comprising 2 to 3 individuals. Due to the scheduling conflicts, 6 parents participated in individual interviews. Interviews were coded using a theoretical thematic analysis approach [33]. Parents reported barriers to engaging in health-promoting behaviors (eg, time constraints, medication side effects, individual preferences, and mood) as well as facilitators to behavior change (eg, family collaboration, goal setting, and monitoring behaviors). Participants also discussed perceived relationships between asthma and obesity and described their intervention preferences (eg, frequency of contact, method of contact, and nurse involvement).

A total of 10 participants have also consented for participation in the PDSs and usability testing, which is expected to conclude in 2019. By the end of the study, we plan to develop a highly usable, useful, easy-to-use mHealth app, mCHAMP, that can enable parents to self-manage their child’s asthma and obesity health-related behaviors. The first results are expected to be submitted for publication in late 2019.

## Discussion

BFIs are the gold standard for promoting effective weight self-management in children [13,14]. Our team recently developed a tailored BFI for children with asthma and obesity. Although results indicated changes in weight and asthma outcomes were promising for completers, attendance was suboptimal and multiple barriers prevented families from attending in-person groups [20]. mHealth is uniquely positioned to address previously observed attendance challenges [28,29], deliver individualized content, and has demonstrated improvements in dietary intake and physical activity in youth with obesity [25]. To our knowledge, this is the first study to develop a nurse-delivered BFI supported by an mHealth app and tailored to the needs of children with comorbid asthma and obesity. Products of this study are expected to include an mHealth app designed by the target audience, initial usability data from stakeholders as well as target users, and a refined nurse-delivered self-management intervention protocol. This study is also expected to demonstrate an innovative approach to integrating multiple intervention development and adaptation frameworks.

## Abbreviations

ADAPT-ITT: Assessment Decision Administration Production Topical Experts Integration Training Testing
BFIs: behavioral family lifestyle intervention
BMI: body mass index
C2PD: consumer-centered participatory design
CHAMP: Childhood Health and Asthma Management Program
EMA: ecological momentary assessment
mCHAMP: Mobile Childhood Health and Asthma Management Program
mHealth: mobile health
PDSs: participatory design sessions
PSHUQ: Post-Study e-Health Usability Questionnaire
UF: University of Florida
